# Myocarditis Elicits Dendritic Cell and Monocyte Infiltration in the Heart and Self-Antigen Presentation by Conventional Type 2 Dendritic Cells

**DOI:** 10.3389/fimmu.2018.02714

**Published:** 2018-11-21

**Authors:** Katrien Van der Borght, Charlotte L. Scott, Liesbet Martens, Dorine Sichien, Gert Van Isterdael, Veronika Nindl, Yvan Saeys, Louis Boon, Burkhard Ludewig, Thierry C. Gillebert, Bart N. Lambrecht

**Affiliations:** ^1^Immunoregulation and Mucosal Immunology, VIB-UGent Center for Inflammation Research, Ghent, Belgium; ^2^Department of Internal Medicine, Ghent University, Ghent, Belgium; ^3^Department of Biomedical Molecular Biology, Ghent University, Ghent, Belgium; ^4^Institute of Immunobiology, Kantonsspital St. Gallen, St. Gallen, Switzerland; ^5^Bioceros, Utrecht, Netherlands; ^6^Department of Pulmonary Medicine, Erasmus MC, Rotterdam, Netherlands

**Keywords:** dendritic cells, myocarditis, autoimmunity, heart failure, autoreactive T cells

## Abstract

Autoimmune myocarditis often leads to dilated cardiomyopathy (DCM). Although T cell reactivity to cardiac self-antigen is common in the disease, it is unknown which antigen presenting cell (APC) triggers autoimmunity. Experimental autoimmune myocarditis (EAM) was induced by immunizing mice with α-myosin loaded bone marrow APCs cultured in GM-CSF. APCs found in such cultures include conventional type 2 CD11b^+^ cDCs (GM-cDC2s) and monocyte-derived cells (GM-MCs). However, only α-myosin loaded GM-cDC2s could induce EAM. We also studied antigen presenting capacity of endogenous type 1 CD24^+^ cDCs (cDC1s), cDC2s, and MCs for α-myosin-specific TCR-transgenic TCR-M CD4^+^ T cells. After EAM induction, all cardiac APCs significantly increased and cDCs migrated to the heart-draining mediastinal lymph node (LN). Primarily cDC2s presented α-myosin to TCR-M cells and induced Th1/Th17 differentiation. Loss of IRF4 in *Irf4*^*fl*/*fl*^*.Cd11cCre* mice reduced MHCII expression on GM-cDC2s *in vitro* and cDC2 migration *in vivo*. However, partly defective cDC2 functions in *Irf4*^*fl*/*fl*^*.Cd11cCre* mice did not suppress EAM. MCs were the largest APC subset in the inflamed heart and produced pro-inflammatory cytokines. Targeting APC populations could be exploited in the design of new therapies for cardiac autoimmunity.

## Introduction

In acute myocarditis immune and inflammatory cells infiltrate the heart, causing damage to cardiomyocytes, transient heart failure and life threatening arrhythmias (1). Whereas cardiac inflammation often disappears spontaneously, a subset of patients progresses to dilated cardiomyopathy (DCM) and chronic heart failure with high mortality (2, 3). Acute myocarditis is traditionally triggered by coxsackie B3 (CVB3) infection that directly destroys cardiomyocytes as part of their replicative lytic cycle (4). As adaptive immunity can be triggered by infection or release of danger associated molecular patterns (DAMPs) from dying cells, there is inherent risk of autoimmunity following acute myocarditis. Organ-directed autoimmunity perpetuates inflammation and is a risk factor for DCM development as a result of continuing myocardial injury (5).

Self-reactive T cells are usually removed during thymic negative selection. However, CD4^+^ T cells directed against cardiac α-myosin that escape this checkpoint, enter the periphery and become part of the normal T cell repertoire in healthy mice and humans (6, 7). To prevent autoimmunity, it is critical to keep these heart-reactive T cells in a quiescent state by mechanisms of peripheral tolerance (8, 9). Infection of the heart with CVB3 virus can however activate α-myosin specific autoreactive CD4^+^ T cells that infiltrate the heart and produce IL-17 (10). To distinguish the infectious from the autoimmune phase, mouse models of experimental autoimmune myocarditis (EAM) have been established in which cardiac-specific T cell activation is initiated by immunization with heart self-antigen in adjuvant systems. In these highly immunogenic models, CD4^+^ T cells are critical for the induction of EAM (11).

Activation of autoreactive CD4^+^ T helper cells is strongly regulated by dendritic cells (DCs). In steady state, DCs maintain peripheral tolerance by rendering autoreactive T cells anergic or converting naïve T cells into induced regulatory T cells (iTreg) (12). However, when tissues are inflamed or when the activation threshold of DCs is genetically altered, DCs activate self-reactive T cells which leads to autoimmunity (13–17). The potential of DCs to initiate cardiac autoimmunity is supported by the ability of LPS-matured bone marrow derived DCs (BMDC) loaded with an immunogenic peptide derived from cardiac α-myosin heavy chain (αMyHC_614−629_) or apoptotic cardiomyocytes to induce EAM in wild type (WT) mice (18–21).

Although the BMDC-induced EAM model pointed to an important role for DCs as inducers of cardiac autoimmunity, it is still unclear what role endogenous heart DCs play in the onset and progression of disease (9). We now realize that there is considerably more heterogeneity in DCs and more overlap with macrophages (MFs) than initially thought, which has led to new classification systems of DCs into conventional DC type 1 (cDC1), conventional DC type 2 (cDC2), plasmacytoid DCs (pDC) and monocyte-derived cells (MCs), which can function as DCs and/or MFs (22–26). Although DCs and MFs are present in the heart of all species studied (27–32), a detailed study of cardiac DC and MC subsets and their implication in autoimmunity is lacking. We have also come to realize that BMDCs such as those used to initiate the EAM model (18–21) are heterogeneous, consisting of both GM-cDCs and GM-MCs (33, 34). Therefore, the precise role of DC subsets and MCs in the initiation and progression of EAM is unclear at present.

Here we investigated the function of endogenous heart APC subsets and their contribution to regulating the heart-specific immune response by using α-myosin-specific CD4^+^ T cell receptor (TCR) transgenic T cells (TCR-M cells) as readout of antigen presenting function (35), and induction of EAM as readout of pathogenic autoimmunity. By carefully sorting GM-cDC and GM-MC subsets from BMDC cultures, we conclusively show that mature GM-cDCs are the main drivers of autoimmune T cell attack and induction of EAM. Endogenous cDC and MC subsets were separated during EAM in heart and draining lymph node (LN) and were used for studying gene expression profiles by RNA sequencing. The transcriptome of cardiac APCs revealed a superior role in self-antigen presentation for cDC2s as was also confirmed by *ex vivo* co-cultures. By using mice that genetically lack the key transcription factor (TF) IRF4 affecting cDC2 function, we show that cDC2s lacking IRF4 can still partially migrate to the mLN and present αMyHC to TCR-M cells. Reduced cDC2 migration has no impact on EAM severity suggesting that the remaining migratory cDC2s are sufficient for sustaining EAM. Endogenous cardiac MCs are potentially required for EAM by producing pro-inflammatory cytokines and chemokines. Thus, interfering with the function and activation of MCs could help in treating or preventing cardiac autoimmunity.

## Materials and methods

### Mice

Wild type (WT) Balb/c mice were purchased from Harlan and Janvier. αMyHC-TCR transgenic mice (TCR-M) on Balb/c background were previously described (35). *Irf4*^*fl*/*fl*^*.Cd11cCre* mice were backcrossed onto the Balb/c background for at least 2 generations.

The age of the mice at use was 5–7 weeks, and mice were housed in SPF conditions. The animal ethics committee of VIB Inflammation Research Center and University Hospital Ghent approved all experiments.

### GM-CSF cultures

Bone marrow cells were freshly isolated from femur and tibia by crushing in RPMI 1640 medium. 3 × 10^6^ bone marrow cells were cultured in petri dishes in 10 ml of tissue culture medium (TCM) (RPMI 1640, glutamax, gentamycin, 2-mercaptoethanol, 5% heat-inactivated fetal calf serum), and GM-CSF (20 ng/ml, in house generated) and placed at 37°C and 20% O2/5% CO2. 10 ml of new TCM was added at day 3 of culture and at day 6 half of the medium was refreshed. BMDCs were harvested on day 10 by collecting the 20 ml of culture medium and washing with 5 ml PBS/EDTA (15 min−37°C) (50 μM). In some experiments, BMDCs were *in vitro* labeled with cell proliferation dye eFluor450 (ebioscience) before intraperitoneal (i.p.) injection.

### Induction of myocarditis

BMDC-induced EAM was performed with minor modifications of an established protocol (19). On day 10 of GM-CSF culture, BMDCs were pulsed with synthetic α-Myosin Heavy Chain peptide (αMyHC_614−629_ at 15 μg/ml) or ovalbumin peptide (OVA_323−339_) (custom made, Pepscan) and matured with 100 ng/ml LPS (ultra-pure lipopolysaccharide from *E. coli* 0111:B4 strain; InvivoGen) and 5 μg/ml of agonistic antibodies (Abs) to CD40 (clone FGK45, Bioceros) for 4 h. To initiate EAM, mice were i.p. injected with 5 × 10^5^ to 1 × 10^6^ BMDCs at day 0, 2, and 4. As a negative control, mice received mature OVA_323−339_ pulsed BMDCs. GM-DCs, and GM-MCs FACS purified from bulk BMDC cultures were also used to induce EAM. After cell sorting, GM-DCs and GM-MCs were antigen loaded and matured for 4 h in complete TCM with GM-CSF (20 ng/ml), αMyHC_614−629_ (15 μg/ml), LPS (100 ng/ml) and αCD40 antibodies (5 μg/ml). WT mice were injected i.p. with 8 × 10^5^ to 1 × 10^6^ GM-DCs or GM-MCs at day 0, 2 and, 4 for priming EAM.

### Tissue digestion

Heart left and right ventricles were flushed with PBS. Organs were manually cut in 0.5 mm pieces using scissors. Samples were enzymatically digested with 20 μg/ml liberase TM research grade (Roche) and 10 U DNase (Roche) in RPMI 1640 medium for 30–45 min at 37°C. Digested tissues were filtered through 70 μm cell strainer to remove undigested tissue and to obtain single cell suspensions. Osmotic lysis buffer was added for 3 min to remove erythrocytes. Tissue cell suspensions were then used as the starting source of material for flow cytometry labeling and cell sorting.

### Flow cytometry labeling and cell sorting

Single cell suspensions from tissues or BMDC cultures were incubated with a mix of fluorescently-labeled monoclonal Abs for 30 min at 4°C. To reduce non-specific binding, 2.4G2 Fc receptor Ab was added. Following Abs were used: anti-CD11c (clone N418), anti-MHCII (clone M5/114), anti-CD11b (clone M1/70), anti-PD-L2 (clone 122), anti-CD40 (clone 3/23), anti-CD80 (clone 16-10A1), anti-CD86 (clone PO3), anti-CD26 (clone H194-112), anti-CD115 (clone AFS98), anti-CCR7 (clone 4B12), anti-CD25 (clone PC61), anti-CD44 (clone IM7), anti-V alpha 2 chain (clone B20.1), anti-V beta 8.1 8.2 chain (clone MR5-2), anti-CD3 (clone 145-2C11), anti-CD4 (clone RM4-5), anti-CD45.2 (clone 104), anti-CD19 (clone 1D3), anti-Ly6C (clone AL-21), anti-Ly6G (clone 1A8), anti-CD64 (clone X54-5/7.1), anti-CD172α (clone P84), anti-XCR-1 (clone ZET), anti-CD8α (clone 53-6.7), anti-CD24 (clone M1/69), anti-T-bet (clone 4B10), anti-RoRγt (clone Q31-378), anti-Foxp3 (clone FJK-16s), anti-IFNγ (clone XMG1.2), anti-IL-17A (clone TC11-18H10), anti-CD317 (clone 120G8), anti-B220 (clone RA3-6B2), anti-IRF4 (clone M-17). To gate out dead cells, we used fixable viability dye eFluor 506 (eBioscience). To detect intracellular cytokines, cells were stimulated with phorbol 12-myristate 13-acetate (PMA, Sigma), ionomycin (Sigma) and Golgistop Protein Transport Inhibitor (BD) for 4–5 h at 37°C. For intracellular cytokine staining, cells were fixed using a fixation/permeabilization kit (eBioscience) according to the manufacturer's protocol. Flow cytometry was performed on a LSR Fortessa cytometer (BD Biosciences). For cell sorting, a FACSAria high-speed sorter (BD Biosciences) was used. Final analysis and graphical output were performed using FlowJo software (Tree Star, Inc.).

### Adoptive transfer of TCR-M cells

Spleens were collected from TCR-M mice and were mechanically disrupted on a 70 μm cell strainer. Naïve TCR-M splenocytes were MACS purified using CD4^+^CD62L^+^ T Cell isolation kit (Miltenyi). TCR-M cells were stained with carboxyfluorescein succinimidyl ester (CFSE, Invitrogen). 1 × 10^6^ naïve TCR-M cells were injected intravenously (iv.) in the lateral tail vein into WT steady state mice. One day later, bulk BMDCs (1 × 10^6^), sorted GM-DCs (2 × 10^5^) or GM-MFs (2 × 10^5^) loaded with OVA or αMyHC were i.p. injected. After 3 days, mice were sacrificed and inguinal LN from the injected and opposite side, axillary LN (injected side), mesenteric LN, mediastinal LN, spleen, and heart were isolated. Individual cell suspensions were prepared by mechanical disruption and stained for flow cytometry.

### Co-cultures of sorted DC subsets and TCR-M cells

For *ex vivo* co-cultures, tissues were collected from steady state mice and mice on day 10 of EAM. DC subsets were sorted as described above. For co-cultures with *in vitro* BMDCs, GM-DCs, and GM-MCs were sorted from bulk BMDC cultures. TCR-M cells were labeled with carboxyfluorescein succinimidyl ester (CFSE, Invitrogen) or with cell proliferation dye eFluor450 (ebioscience) to track their proliferation. 5 × 10^4^ labeled TCR-M CD4^+^ T cells were added to the sorted DC subsets in a 1 in 5 DC/T cell ratio in complete TCM medium. In some wells 15 μg/ml of αMyHC614-629 or dead cardiomyocytes were added to the medium. To obtain cardiomyocytes, CD45^+^ leukocytes were removed by CD45 MACS positive selection (Miltenyi) from a single cell suspension of steady state heart. Per well, 5% of 1 total digested heart was added. CFSE dilution and T cell activation were evaluated by flow cytometry after 3–4 days. IL-17A and IFNγ ELISA (eBioscience, Ready-Set-Go kit) was performed on the co-culture cell supernatants (collected on day 3–4) according to the manufacturer's protocol.

### Real-time PCR

DC subsets were sorted from at least 10 pooled hearts on day 10 of the EAM protocol. Sorted cells were pelleted and suspended in TriPure isolation reagent (Roche). RNA extraction was performed following manufacturer's protocol. Transcriptor high fidelity cDNA synthesis kit with random hexamer primers (Roche) was used to reverse transcribe 500 ng RNA into cDNA. cDNA was diluted 6–17,5 times in ultrapure water for use in RT-PCR reactions. RT-PCR primers for used targets have been described (36). RT-PCR reaction was performed with sensiFAST Sybr no-ROX mix (Bioline) and was performed in triplicate in a Roche LCII 480. Relative expression of targets was calculated by comparison to HPRT and SDHA expression in qBase+ software (Biogazelle, Gent, Belgium).

### RNA sequencing

cDC1s, cDC2s, CD11c^+^ MC, and CD11c^−^ MCs were sorted by FACS from 100 pooled steady state and 12 pooled inflamed hearts (EAM day 10) of female WT Balb/c mice. 20,000 cells were sorted straight into 500 μl buffer (RLT Plus; Qiagen) and 5 μl β-mercaptoethanol and RNA was isolated by a micro-RNA isolation kit (Qiagen). RNA was amplified by SmarTER amplification (Clontech) because RNA amounts were low. RNA sequencing was performed at the Nucleomics facility (VIB, Leuven) using NextSeq sequencer (Illumina). Trimmomatic was used for the preprocessing of the data and adapters were cut off. Reads were clipped when the quality dropped below 20 and were excluded when longer than 35. FastQC was performed and all samples passed quality control. Reads were plotted to the mouse reference genome via Tophat2 and calculated via HTSeqCount. R/Bioconductor was utilized to analyze the samples and data normalization was performed using the DESeq2 procedure. To identify unique cDC2 genes we filtered the normalized log2 expression table using the R package “sqldf”. Log2 expression of cDC2s from MI heart needed to be at least 1.2 log2 value bigger than cDC2s from steady state heart. The differences between the other steady state and EAM samples needed to be lower than 0.3 log2 value.

### Ultrasound

Transthoracic echocardiography was performed using a 38 MHz probe and a Vevo 2100 Ultrasound machine (Visualsonics). The mice's chests were shaved using VEET and mice were lightly anesthetized with 0.7–1.5% isoflurane. Heart rate was conserved at 400–500 beats per min. At least three M-mode images of the short axis at mid-ventricular level were recorded. Diastolic and systolic internal ventricular septum thickness (IVS, d and IVS, s), diastolic and systolic left ventricular posterior wall thickness (LVPW, d and LVPW, s) and left ventricular end-diastolic and end-systolic internal diameter (LVID, d and LVID, s) were measured using Vevo 2100 1.5.0 software. Per short axis M-mode image, dimensions of three cardiac cycles were averaged. In total, three independent M-mode images were used for heart function measurements.

### Statistical analysis

For all experiments, the difference between groups was calculated using the Mann-Whitney *U*-test for unpaired data (GraphPad Prism version 6.04, San Diego, CA). Differences were considered significant when *P* < 0.05.

## Results

### Transfer of bone marrow cDC2s initiates EAM

**Figure 1:**
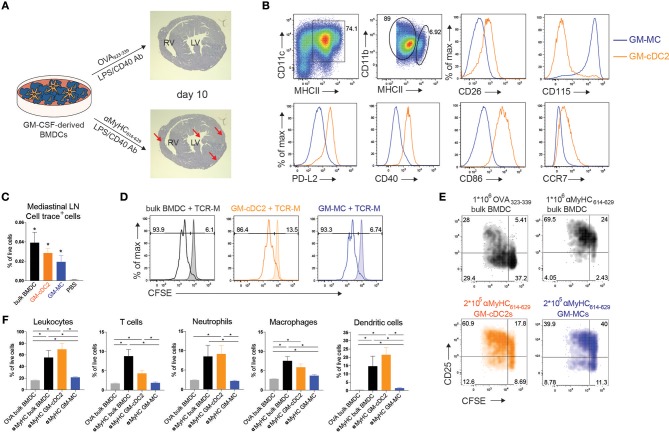
Transfer of bone marrow cDC2 initiates EAM. **(A)** Schematic overview of BMDC-induced mouse model of experimental autoimmune myocarditis (EAM). GM-CSF derived BMDCs were matured using LPS and αCD40 and loaded with heart specific self-antigen αMyHC_614−629_ or the harmless OVA_323−339_ antigen. αMyHC_614−629_ BMDC immunization leads to fulminant myocarditis (bottom, red arrows) as shown by heamatoxylin and eosin staining of heart sections at day 10 post-immunization, whereas OVA BMDC immunization does not (top). RV, Right Ventricle; LV, Left Ventricle. **(B)** Flow cytometric analysis of GM-CSF derived BMDCs harvested at day 10 of culture. Histograms display expression of the specified markers by MHCII^int^ GM-MCs and MHCII^hi^ GM-cDC2s. **(C)** Cell trace^+^ cell percentages of total living cells in mLN of i.p. injected mice with 0.5 × 10^6^ cell trace-labeled BMDCs, GM-cDC2s, GM-MCs, or PBS 12 h before sacrifice. **(D)** Histograms representing CFSE dilution of TCR-M cells co-cultured with bulk BMDCs, sorted GM-cDC2s, or sorted GM-MCs with or without 15 μg/ml αMyHC_614−629_ (respectively, non-filled and filled histograms) in a 1/5 DC/TCR-M ratio. **(E)** 4 days after naïve TCR-M injection and 3 days after OVA-loaded BMDC, αMyHC-loaded BMDC, GM-cDC2, or GM-MC injection, mediastinal LN was isolated. CFSE dilution and CD25 expression of donor TCR-M cells was analyzed by flow cytometry. **(F)** Leukocyte, T cell, neutrophil, macrophage and DC percentages of total living cells in the heart of OVA BMDCs, αMyHC BMDC, αMyHC GM-cDC2, and αMyHC GM-MC injected mice at day 10 post-injection. All bar graphs in this figure shows data as the mean ± SEM; ^*^P ≤ 0.05.

EAM was initiated in WT Balb/c mice by immunization with *in vitro* GM-CSF-derived bulk BMDCs loaded with cardiac αMyHC_614−629_ peptide according to a published protocol (Figure 1A) (19). BMDCs were matured by TLR4 stimulation and CD40 triggering. Only mice receiving three intraperitoneal (i.p.) αMyHC_614−629_-loaded BMDC injections (day 0, 2, and 4) but not those receiving control OVA_323−339_ peptide pulsed BMDCs developed severe EAM characterized by massive and patchy infiltration of leukocytes in the heart 10 days after the first immunization (Figure 1A). In some patients, the initial phase of autoimmune myocarditis can progress to DCM. In our hands, immunization with αMyHC via BMDCs elicits a mild DCM with dilation of the left ventricle in systole (LVID, sys) (Supplementary Figures 1A,B), decrease in ejection fraction (EF) (Supplementary Figure 1C) and increase in heart/body weight ratio (Supplementary Figure 1D) without high mortality (Supplementary Figure 1E).

Recently it was observed that CD11c^+^MHCII^+^ BMDCs grown in GM-CSF are not a uniform population but consist of conventional DCs (GM-cDCs) and monocyte-derived cells (GM-MCs) with a macrophage-like phenotype in C57Bl/6 mice (34). We found the same heterogeneity in Balb/c BMDC cultures (Figure 1B). CD11c^+^MHCII^int^ cells expressed higher amounts of the macrophage marker CD115 (receptor for Macrophage Colony-Stimulating Factor) and were named GM-MCs, whereas MHCII^hi^ cells expressed the cDC specific marker CD26 (24) and were termed GM-cDCs. These GM-cDCs also expressed CD172α, high levels of the transcription factor IRF4, and low levels of IRF8 (Supplementary Figure 2A) suggesting they were of the cDC2 lineage and were termed GM-cDC2s to stress their cDC2 phenotype. GM-cDC2s were in a more mature state compared to GM-MCs as demonstrated by their higher surface expression of PD-L2, CD40, CD86 and CCR7 (Figure 1B). It is unclear if GM-cDC2s and GM-MCs have distinct ability to induce EAM. Similar to bulk BMDCs, 12 h after i.p. injection, fluorescently labeled, and sorted GM-cDC2s and GM-MCs (Supplementary Figure 2A) both migrated to the mediastinal LN (mLN) (Figure 1C), the LN draining the peritoneum, heart and lung (37, 38). To probe for antigen presenting capacity, GM-cDC2s, and GM-MCs were loaded with αMyHC_614−629_ peptide, placed in co-culture with CFSE labeled TCR-M cells, where both showed similar capacity to induce proliferation (Figure 1D).Peptide can readily bind to MHCII on APCs, therefore the uptake and processing of dead cardiomyocytes by GM-cDC2s and GM-MCs was also tested. Both GM-cDC2s and GM-MCs induced proliferation of TCR-M cells when dead cardiomyocytes were added to the co-culture with labeled TCR-M cells (Supplementary Figure 2B). GM-MCs were even slightly better than GM-cDC2s in activating TCR-M cells. These data suggest that the different cell types in the GM-CSF BMDC cultures have comparable antigen processing and presenting capacities at least *in vitro*.

To verify the ability of i.p. injected BMDC subsets to prime TCR-M cells *in vivo*, we transferred naïve CFSE^+^ TCR-M cells into WT mice 1 day prior to i.p. BMDC administration. After 3 days, TCR-M cells proliferated in the mLN of non-injected (Supplementary Figure 2E) and OVA BMDC injected mice (Supplementary Figures 2C,D) showing that endogenous αMyHC is presented in the heart-draining mLN without any cardiac damage (8, 35). Nevertheless, mice injected with αMyHC-loaded BMDCs displayed more TCR-M activation in mLN (Figure 1E) due to the additional presentation of αMyHC on injected BMDCs. Some bystander activation of TCR-M cells was observed in mesenteric LN, spleen and inguinal LN at injection side, but only limited proliferation was detected (Supplementary Figures 2C,D). I.p. injection of myosin peptide loaded GM-cDC2s and GM-MCs induced greater CD25 expression on TCR-M cells than OVA loaded BMDCs (Figure 1E). However, GM-MCs induced less proliferation than GM-cDC2s and bulk BMDCs *in vivo*. Lastly, we evaluated the ability of GM-cDC2s and GM-MCs to elicit EAM *in vivo*. Immunization with αMyHC-loaded GM-MCs failed to induce severe EAM whereas αMyHC-loaded GM-cDC2s triggered EAM at levels comparable with αMyHC bulk BMDCs, reflected by the presence of various inflammatory cells in the heart (Figure 1F). Thus, within GM-CSF cultured bulk BMDCs, GM-cDC2s have the potency to initiate EAM whereas GM-MCs do not.

### DC subsets accumulate in heart and mLN during EAM

Data about the functional contribution of endogenous counterparts of the bone marrow GM-cDC2s and GM-MCs in progression of EAM are currently lacking. cDCs are divided into cDC1s and cDC2s that arise from a DC-committed hematopoietic progenitor. cDC1s depend on transcription factor IRF8 (39) and are superior at cross-presenting exogenously acquired antigens to CD8^+^ T cells (40). Tissue cDC2s depend partly on IRF4 (41), require IRF4 for migration to the LN and are specialized in presenting antigens in the context of MHCII to CD4^+^ T cells (42). Monocytes entering tissues can start to express CD11c and MHCII and take on DC functions, for which they were historically named monocyte-derived DCs (moDCs), although recent lineage tracing strategies have shown that these cells also closely resemble MFs (25, 43).

**Figure 2:**
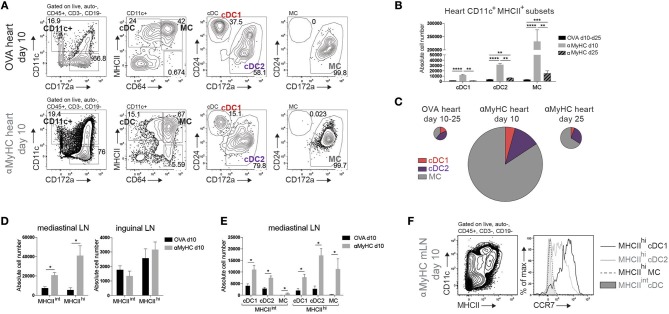
DC subsets accumulate in heart and mLN during EAM. **(A)** Representative flow cytometry gating strategy for cDCs, CD11c^+^ MCs, cDC1s, and cDC2s in the heart of OVA or αMyHC BMDC injected WT mice on day 10 post-injection. Numbers specify cell percentage inside nearby gates. **(B)** Absolute cell numbers of cDC1s, cDC2s, and MCs in the heart of OVA (pooled data of day 10 and day 25 post-inj.) and αMyHC BMDC (at day 10 and day 25 post-inj.) injected WT mice as assessed by flow cytometry. **(C)** Pie charts depicting the distribution of APC subsets in heart of OVA (pooled data of day 10 and day 25 post-inj.) and αMyHC BMDC (at day 10 and day 25 post-inj.) injected WT mice. Pie chart sizes are proportional to total absolute cardiac APC numbers. **(D,E)** Absolute numbers of MHCII^int^ and MHCII^hi^ DCs **(D)** and MHCII^int^ and MHCII^hi^ subsets **(E)** in the heart-draining mediastinal LN **(D,E)** and skin-draining inguinal LN **(D)** of OVA and αMyHC BMDC injected WT mice at day 10 post-injection. **(F)** Illustrative flow cytometry gating strategy for MHCII^int^ and MHCII^hi^ CD11c^+^ cells and CCR7 expression of MHCII^hi^ cDC1s, MHCII^hi^ cDC2s, MHCII^hi^ MCs, and MHCII^int^ cDCs in mLN of αMyHC BMDC injected WT mice at day 10. All bar graphs in this figure shows data as the mean ± SEM; ^*^P ≤ 0.05; ^**^P ≤ 0.01; ^***^P ≤ 0.001; ^****^P ≤ 0.0001.

Cardiac CD11c^+^ cells were identified amongst live non-autofluorescent CD45^+^ Lineage^−^ cells (Figure 2A). cDCs were separated from MCs by the MF marker CD64. cDCs were further subdivided into CD24^+^ cDC1s and CD172α^+^ cDC2s and MCs uniformly expressed CD172α. 10 days after immunization, all cDC, and MC subsets increasingly infiltrated the heart of αMyHC BMDC injected mice compared with OVA controls (Figures 2A–C). MCs contributed 84% of CD11c^+^MHCII^+^ cells in EAM day 10 heart (Figure 2C). During the DCM phase (day 25), cDC1s returned to baseline levels while cDC2s and MCs remained elevated compared to OVA-BMDC injected mice (Figure 2B).

To prime naïve autoreactive CD4^+^ T cells, heart-resident DCs need to migrate in a chemokine receptor CCR7-dependent manner to the draining mLN to present self-antigen, and in this process upregulate MHCII expression. MHCII^hi^ migratory and MHCII^int^ LN-resident cDCs and MCs were increased in mLN at EAM day 10 (Figure 2D). Increase of these cells was exclusive to the mLN as DCs in skin draining inguinal LN remained stable (Figure 2D). All subsets of cDCs and MCs were enriched in mLN but MHCII^hi^ cDC2s were the largest subset (Figure 2E). Migratory MHCII^hi^ cDC1s and cDC2s expressed CCR7 while MHCII^int^ LN-resident cDCs lacked CCR7. In contrast, MHCII^hi^ MCs lacked CCR7 expression showing that they had most likely not migrated from heart to mLN, but rather reached the mLN via the bloodstream while upregulating MHCII (44) (Figure 2F). We conclude that endogenous CD11c^+^MHCII^+^ APC subsets infiltrate the heart and cDCs actively migrate from the inflamed heart to the draining LN in EAM.

### Expression profiling of cardiac APCs by RNA sequencing

**Figure 3:**
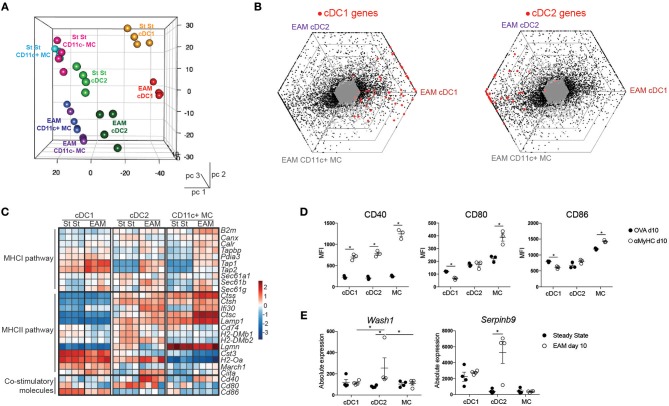
Expression profiling of cardiac APCs by RNA sequencing. **(A)** Front view of PCA of RNA-seq data from cDC1s, cDC2s, CD11c+ MC, and CD11c- MCs in the steady state (St St) and the inflamed (EAM d10) heart. PCA was calculated using the top 15% most varying genes between cell subsets. Each dot symbolizes one independently sorted replicate of the indicated cell population and four independent sorts were performed per subset. **(B)** Triwise diagrams of the gene expression at EAM day 10. Red dots represent cDC1 (left) and cDC2 hallmark genes (right). **(C)** Heatmap showing the relative expression of MHCI- or MHCII-associated genes normalized per mean expression of each gene in cardiac cDC1s, cDC2s, and CD11c+ MCs from steady state and EAM day 10 heart. **(D)** Mean Fluorescence Intensity of CD40, CD80, and CD86 expression on heart DC subsets in OVA and αMyHC BMDC injected WT mice at day 10 post-injection. **(E)** Graphs representing absolute expression of *Wash1* and *Serpbinb9*, two cDC2-specific DEGs between steady state and EAM day 10 assessed by RNA-seq. All bar graphs in this figure show data as the mean ± SEM; ^*^P ≤ 0.05.

To further characterize APC subsets in the inflamed heart, cDC, and MC subsets were sorted 10 days post immunization and were subjected to RNA-sequencing (RNA-seq) analysis. For comparison, we also sorted CD11c^−^CD64^+^MHCII^+^ cardiac MF-like MCs. By performing a principal component analysis (PCA), we observed that PC1 corresponded to ontogeny of the APCs whereas PC2 corresponded to the inflammatory milieu in the EAM heart (steady state vs. EAM) (Figure 3A). Triwise comparisons of gene expression (45, 46) revealed that expression of hallmark cDC1 genes was conserved in cDC1s from EAM hearts. Many hallmark cDC2 genes (45) were shared between cardiac cDC2s and MCs in EAM mice (Figure 3B). Given that cDCs are professional APCs, we evaluated the expression profile of genes involved in the MHCI or MHCII pathway and genes linked to T cell co-stimulation in heart cDCs and MCs in steady state and EAM (day 10) (Figure 3C). In EAM, all heart APC subsets gained expression of genes involved in the MHCI pathway (*Tapbp, Tap1, Tap2, Sec61b, Sec61g*). However, cDC1s had highest expression of *Tap1* and *Tap2* suggesting that they were better in cross-presenting endogenously acquired antigens to CD8^+^ T cells via the MHCI pathway. Similarly, some genes linked to the MHCII pathway of antigen presentation including *Ifi30, Ctsc*, and *Ciita* were increased in cDC2s and MCs from EAM heart compared with steady state. In cDC1s from inflamed hearts, only expression of *Ciita* increased. Fitting with cDC2s being superior in presentation to CD4^+^ T cells via the MHCII pathway, heart cDC2s displayed high expression of most MHCII related genes in steady state and EAM (*Ctss, Ctsh, Ifi30, Ctsc, Lamp1, Cd74, H2-DMb1, H2-DMb2, H2-Oa, Ciita*) (42). Mature cDCs express surface co-stimulatory molecules to efficiently activate naïve T cells. We therefore evaluated the RNA levels of *Cd40, Cd80*, and *Cd86* and observed that *Cd40* expression indeed increased on cDC2s and MCs in EAM compared with steady state. On protein level, CD40 surface expression increased on all subsets while CD80 and CD86 were only increased on MCs in EAM hearts suggesting that all APCs but especially MCs in the inflamed heart are in an activated state (Figure 3D). To identify interesting genes, we assessed which genes were differentially expressed in cDC2s from steady state compared to EAM heart and not in the other APC subsets. We found 41 cDC2 specific DEGs (Supplementary Table 1). *Wash1* (WAS protein family homolog (1) in DCs is necessary for priming of autoreactive T cells (47) and *Serpinb9* (serine peptidase inhibitor clade B member (9) is linked to DC maturation and protects DCs from the kiss of death by cytotoxic CD8^+^ T cells (48, 49) (Figure 3E). In total, 5 out of 41 genes have a clear role in the function of DCs and 7 genes are involved in several immune functions (Supplementary Table 1). The remaining 29 genes have different roles in cell migration and division, metabolism, ciliogenesis or do not have a clear role in DC function or no known molecular function at all. Thus, gene analysis suggested that cardiac cDC2s would be superior in stimulating autoreactive T cells and would have an increased activation state and prolonged survival in the inflamed heart.

### cDC2s present self-antigen in heart and draining LN in EAM

**Figure 4:**
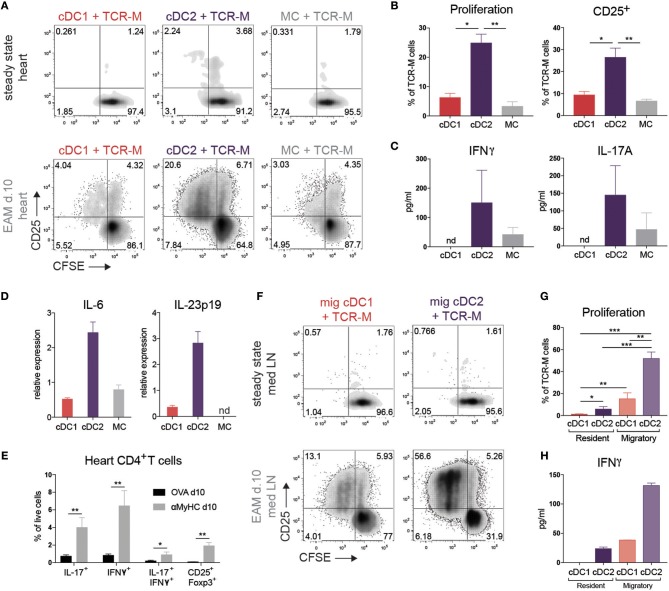
cDC2s present self-antigen in heart and draining LN in EAM. **(A)** CFSE dilution and CD25 expression of TCR-M cells co-cultured for 3 days with sorted heart DC subsets at steady state and at EAM day 10. Hearts were pooled from 100 steady state mice and 6 mice suffering from acute EAM. Data are representative for three independent experiments. **(B)** Percentage of proliferation and CD25 expression of TCR-M cells from co-cultures plotted in panel A (mean ± SEM; ^*^P ≤ 0.05; ^**^P ≤ 0.01). **(C)** After 3 days of co-culture (plotted in **A**), IFNγ and IL-17A (pg/ml) was measured in cell supernatant by ELISA. ELISA was performed in duplo on the same sample (mean ± SD). **(D)** IL-6 and IL-23p19 mRNA expression in DC subsets sorted from at least 10 pooled hearts at EAM day 10 measured by qPCR. Relative mRNA expression was calculated by normalization to housekeeping genes and qPCR reactions were performed in triplicate (mean ± SD). **(E)** Percentages of heart-infiltrated IL-17^+^, IFNγ^+^, IL-17^+^/IFNγ^+^, and CD25^+^Foxp3^+^ CD4^+^ T cells of OVA (black bars) and αMyHC BMDC injected mice (gray bars) 10 days following BMDC injection measured by flow cytometry after PMA/ionomycin restimulation (mean ± SEM; ^*^P ≤ 0.05; ^**^P ≤ 0.01). **(F)** CFSE dilution and CD25 expression of TCR-M cells co-cultured for 3 days with sorted DC subsets of mLN at steady state and at EAM day 10. Data are representative for five independent experiments. **(G)** Percentage of proliferation of TCR-M cells from co-cultures plotted in **(F)** (mean ± SEM; ^*^P ≤ 0.05; ^**^P ≤ 0.01; ^***^P ≤ 0.001). **(H)** After 3 days of co-culture (plotted in **F**), IFNγ (pg/ml) was measured in cell supernatant by ELISA. ELISA was performed in duplo on the same sample (mean ± SD).

To verify if cDC2s were superior in MHCII presentation to CD4^+^ T cells as proposed by RNA-seq analysis, we FACS purified cDC subsets and MCs from steady state or inflamed heart (EAM d10) and cultured these with CFSE^+^ labeled CD4^+^ TCR-M cells in the absence of exogenously added αMyHC peptide. After 4 days of co-culture, none of the DC subsets from steady state heart induced TCR-M proliferation, although TCR-M cells co-cultured with cardiac cDC2s were slightly activated as demonstrated by up-regulation of CD25, suggestive of steady state self-antigen presentation (Figure 4A). Heart cDC2s from inflamed EAM induced the highest degree of proliferation and T cell activation (Figures 4A,B), whereas cDC1s and MCs were less efficient at doing this. The cDC2s of inflamed EAM hearts also induced most IFNγ and IL-17A cytokine production in TCR-M cells (Figure 4C). Fitting with the IL-17A production from TCR-M cells co-cultured with cDC2s, cDC2s expressed the highest levels of the Th17 promoting cytokines IL-6 and IL-23p19 compared with cDC1s and MCs (Figure 4D). In the inflamed heart *in vivo*, IL-17 and IFNγ producing CD4^+^ T cells infiltrated the tissue (Figure 4E), while double producing IL-17A and IFNγ CD4^+^ T cells and CD25^+^Foxp3^+^ Tregs also increased in the heart in acute EAM.

Upon recognizing peripheral antigens and danger signals released from injured cells, APCs mature and migrate to the organ-draining LN and present the antigen to T cells. The mLN is the primary heart-draining LN (Figure 2D) so we next set out to study if APC subsets transport the self-antigen αMyHC to mLN and present it to autoreactive TCR-M cells. None of the APC subsets sorted from the mLN of control mice were able to stimulate TCR-M cells (Figure 4F). On the other hand, migratory cDC2s from EAM mLN induced considerable TCR-M proliferation and activation, suggestive of a superior *ex vivo* capacity of cDC2s in inducing CD4^+^ T cell activation (50) (Figures 4F,G). Although migratory cDC1s and resident cDC2s from EAM mLNs were weak at inducing TCR-M proliferation compared with migratory cDC2s, proliferation was still above the background seen after stimulation with APC subsets from healthy mice (Figures 4F,G). Migratory cDC2s from mLN at EAM d10 induced most IFNγ in TCR-M cells compared to the other mLN DC subsets (Figure 4H). Finally, we verified that all sorted mLN and heart APC subsets had antigen-presenting capacities as they all induced TCR-M proliferation upon addition of synthetic αMyHC_614−629_ peptide to the co-cultures, suggesting that viability was not compromised by the isolation and sorting procedure (Supplementary Figures 3A,B). Neither resident nor migratory cDC subsets from skin-draining inguinal LN at EAM d10 stimulated TCR-M cells (Supplementary Figure 3C). Thus, cDC2s sorted from the heart and draining mLN in acute EAM are superior in presenting the cardiac self-antigen αMyHC *ex vivo* to autoreactive TCR-M cells inducing TCR-M proliferation and Th1/Th17 skewing.

### Loss of IRF4 in CD11c^+^ cells does not affect EAM severity

**Figure 5:**
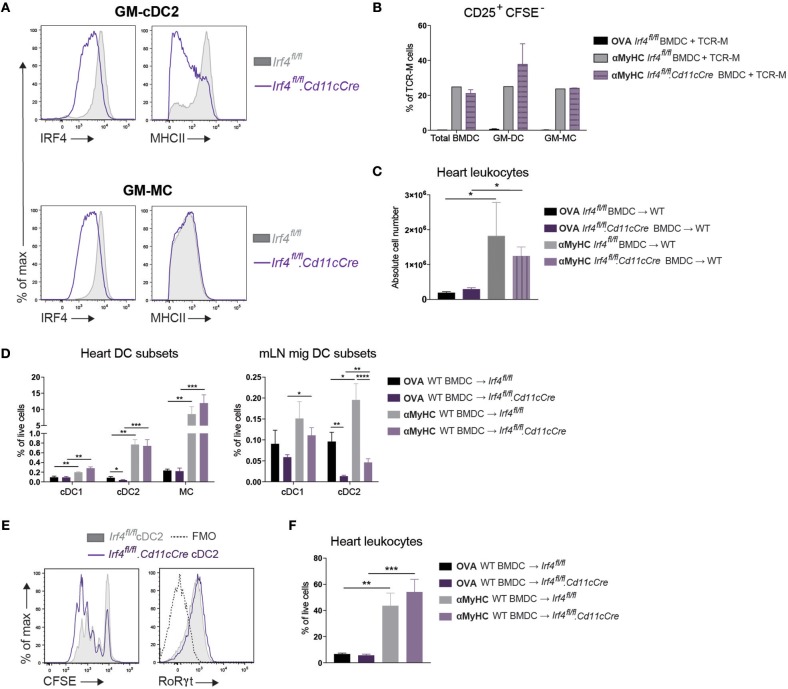
Loss of IRF4 in CD11c+ cells does not affect EAM severity. **(A)** Histograms representing IRF4 and MHCII expression on GM-cDC2s and GM-MCs from *Irf4*^*fl*/*fl*^ and *Irf4*^*fl*/*fl*^*.Cd11cCre* BMDC cultures. **(B)** CD25^+^CFSE^−^ TCR-M cell percentages of total TCR-M cells in co-culture with bulk BMDCs, sorted GM-cDC2s, and sorted GM-MCs from *Irf4*^*fl*/*fl*^ and *Irf4*^*fl*/*fl*^*.Cd11cCre* BMDC cultures with OVA_323−339_ peptide as negative control or with αMyHC_614−629_ peptide. **(C)** Absolute CD45^+^ leukocyte number in the heart of WT mice immunized with OVA_323−339_ or αMyHC_614−629_ loaded BMDCs cultured from *Irf4*^*fl*/*fl*^ and *Irf4*^*fl*/*fl*^*.Cd11cCre* BM. **(D)** APC subset percentages of total living cells in the heart and mLN of *Irf4*^*fl*/*fl*^ and *Irf4*^*fl*/*fl*^*.Cd11cCre* mice immunized with OVA or αMyHC BMDCs (day 10) **(E)** Histograms of CFSE dilution and RoRγt expression of TCR-M cells co-cultured for 4 days with sorted migratory cDC2s from mLN of *Irf4*^*fl*/*fl*^ and *Irf4*^*fl*/*fl*^*.Cd11cCre* mice 10 days after injection with αMyHC BMDCs. **(F)** CD45^+^ leukocyte percentage of total living cells in the heart of *Irf4*^*fl*/*fl*^ and *Irf4*^*fl*/*fl*^*.Cd11cCre* mice immunized with OVA or αMyHC BMDCs (day 10). All bar graphs in this figure shows data as the mean ± SEM; *P ≤ 0.05; **P ≤ 0.01; ***P ≤ 0.001; ****P ≤ 0.0001.

Since we observed that activation of TCR-M cells was primarily initiated by cDC2s *ex vivo*, we explored if cDC2s were necessary for EAM progression in the BMDC-induced model *in vivo*. We opted to use the *Irf4*^*fl*/*fl*^*.Cd11cCre* mice which were previously shown to have reduced priming of CD4^+^ T cells and defective cDC2 migration from lungs and skin to LNs (41) and a defect in Th17 cell differentiation in the gut (50, 51). First, we cultured BMDCs from *Irf4*^*fl*/*fl*^*.Cd11cCre* bone marrow to delineate the role of IRF4 in GM-cDC2s and in EAM induction. CD11c Cre efficiently floxed out *Irf4 in vitro* as demonstrated by decreased IRF4 expression in GM-cDC2s and GM-MCs (Figure 5A). In agreement with literature (42), *Irf4*^*fl*/*fl*^*.Cd11cCre* GM-cDC2s displayed a drop in MHCII expression (Figure 5A). Despite lower MHCII expression, *Irf4*^*fl*/*fl*^*.Cd11cCre* GM-cDC2s could still present αMyHC_614−629_ peptide to CD4^+^ TCR-M cells *in vitro* as efficient as *Irf4*^*fl*/*fl*^ GM-cDC2s (Figure 5B). Immunization with mature αMyHC loaded *Irf4*^*fl*/*fl*^*.Cd11cCre* BMDCs into WT mice resulted in similar leukocyte infiltration in the heart compared with *Irf4*^*fl*/*fl*^ BMDCs (Figure 5C). Thus, loss of IRF4 in CD11c^+^ BMDCs did not affect autoreactive T cell activation *in vitro* and subsequent EAM induction *in vivo*.

Next, *Irf4*^*fl*/*fl*^and *Irf4*^*fl*/*fl*^*.Cd11cCre* mice were immunized with αMyHC loaded WT BMDCs. IRF4 was not required for the accumulation of cDC2s in inflamed hearts (Figure 5D). As observed in other organs, migratory cDC2s were significantly decreased in the mLN of both OVA- and αMyHC-BMDC immunized *Irf4*^*fl*/*fl*^*.Cd11cCre* mice (Figure 5D). However, about 25% of migratory cDC2s persisted in *Irf4*^*fl*/*fl*^*.Cd11cCre* mLN and we addressed if these cDC2s could still present αMyHC *ex vivo*. Therefore, cDC2s were sorted from *Irf4*^*fl*/*fl*^ and *Irf4*^*fl*/*fl*^*.Cd11cCre* mLN (EAM d10) and cultured with TCR-M cells. Proliferation and activation of TCR-M cells was induced to the same degree by *Irf4*^*fl*/*fl*^ and *Irf4*^*fl*/*fl*^*.Cd11cCre* migratory cDC2s (Figure 5E). Similarly, expression of RoRγt, the crucial transcription factor for Th17 cells, was equally high on TCR-M cells stimulated by cDC2s of either genotype. Finally, we evaluated EAM severity and found that *Irf4*^*fl*/*fl*^*.Cd11cCre* mice immunized with αMyHC BMDCs had similar CD45^+^ leukocyte infiltration in the heart (day 10) compared with WT littermates (*Irf4*^*fl*/*fl*^) (Figure 5F). Thus, loss of IRF4 in CD11c^+^ cells has no impact on heart inflammation in the EAM model.

### Cardiac MCs display both DC and MF functions in EAM

**Figure 6:**
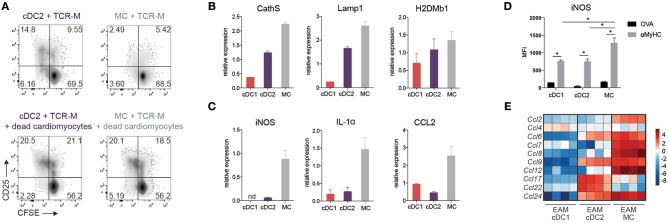
Cardiac MCs display both DC and MF functions in EAM. **(A)** CFSE dilution and CD25 expression of TCR-M cells co-cultured for 3 days with sorted heart cDC2s and CD11c^+^ MCs without and with addition of dead cardiomyocytes. Data are representative for two independent experiments with at least 10 mice per experiment. **(B)** CathS, Lamp1 and H2DMb1 and **(C)** iNOS, IL-1α and CCL2 mRNA expression in cDC1s, cDC2s, and MCs sorted from at least 10 pooled hearts at EAM day 10 measured by qPCR. Relative mRNA expression was calculated by normalization to housekeeping genes and qPCR reactions were performed in triplicate. nd, not detectable. (Mean ± SD) **(D)** MFI of intracellular iNOS expression on heart cDC subsets and MCs in OVA and αMyHC BMDC injected WT mice at day 10 post-injection (mean ± SEM; ^*^P ≤ 0.05). **(E)** Heat map of relative expression of CCL-chemokine genes in cDC1s, cDC2s, and CD11c^+^ MCs sorted from EAM day 10 hearts.

The intact induction of EAM in *Irf4*^*fl*/*fl*^*.Cd11cCre* mice could be due to residual cDC2 function, but could also be due to redundancy with other DCs or MCs. MCs are by far the largest APC subset in the inflamed heart, and share many genes with cDC2s (Figure 3). Therefore, we performed an in depth functional analysis of this subset. As MCs express MHCII molecules, they might be important APCs in the heart. However, compared to cDC2s, MCs of the heart were less capable of inducing TCR-M proliferation *ex vivo* (Figures 4A, 6A). To probe for the full potential to present cardiac antigen upon injury to the heart, dead cardiomyocytes were added to co-cultures of sorted heart cDC2s or MCs (EAM d.10) with TCR-M cells. Whereas freshly purified heart cDC2s readily presented αMyHC to TCR-M cells (Figure 6A), T cell activation and proliferation increased upon addition of dead cardiomyocytes. When dead cardiomyocytes were added to heart MCs, they could fully activate TCR-M cells as well (Figure 6A). In support of a competent antigen presentation capacity, MCs and cDC2s expressed similar amounts of cathepsin S (CathS), lysosomal-associated membrane protein-1 (Lamp1) and the peptide exchanger H2DMb1, factors typically important for presentation via MHCII (Figure 6B).

As in other organs, MCs are poorly migratory and might promote inflammation via chemokine and cytokine production (36). Sorted heart MCs (EAM d.10) strongly expressed pro-inflammatory C-C chemokine ligand 2 (CCL2) and IL-1α, whereas neither cDC subsets did (Figure 6C) providing further evidence that MCs play a role in EAM pathogenesis. Inducible nitric oxide synthase (iNOS), a marker of M1 polarized macrophages, was also highly expressed in MCs compared with cDC subsets both at the level of mRNA and protein (Figures 6C,D). We also looked at a more general set of chemokine genes, and found that MCs expressed the highest amount of mRNA of several CCL-chemokines compared with cDC subsets at EAM day 10 (Figure 6E). These findings suggest a previously unappreciated role for MCs in EAM pathogenesis.

## Discussion

Cardiac damage induced by viral myocarditis, myocardial infarction (MI) or cardiotoxic drugs, can be followed by autoimmunity via activation of autoreactive CD4^+^ T cells. Autoimmunity has a potential to perpetuate the inflammatory response, which promotes progression of disease (4, 11, 19). In many organs, DCs are considered essential for activation of autoreactive CD4^+^ T cells, particularly when DCs are activated by an inflammatory environment caused by infection or necrosis (8, 52). It was previously shown that cardiac myosin is presented by heart-resident MHCII^+^ antigen presenting cells (APC) in the normal mouse heart. EAM augmented the expression of myosin-MHCII complexes in the inflamed heart and boosted APC function (53). In these early studies, APCs were poorly defined and the read-out for antigen presentation was with T cell hybridomas, making it hard to conclude if heart APCs also activate naïve T cells. Also MCs have been shown to infiltrate the heart in CFA/αMyHC induced EAM (31) but the infiltration of cDCs was not verified. Recently, there has been great progress in unraveling the heterogeneity, origin and function of DCs and MCs (22, 23). It was found that *in vitro* cultured BMDCs of C57BL/6 mice using GM-CSF are heterogeneous and contain cells resembling cDCs as well as MCs that more closely resemble MFs (34). Here, in Balb/c mice, we found the same degree of heterogeneity and two clearly distinguishable populations of GM-cDCs and GM-MCs within BMDC cultures. Despite comparable migration and self-antigen presentation of GM-cDCs and GM-MCs, only GM-cDCs from these BM cultures had the potential to induce EAM. Compared with GM-MCs, GM-cDCs are superior in processing and presenting OVA protein to OT-II cells (34). In contrast to these findings, we did not observe a difference between GM-cDCs and GM-MCs in processing and presenting self-antigen derived from dead cardiomyocytes to autoreactive T cells *in vitro* (Supplementary Figure 2B). This discrepancy could be due to the difference in antigen and TCR transgenic T cells used in our experiments. GM-cDCs do have a higher expression of the maturation markers CD40, CD86, and CCR7 (Figure 1B). Injected αMyHC-loaded GM-cDCs induced more TCR-M activation in the mLN *in vivo* (Figure 1E). We argue that GM-cDCs might likewise prime endogenous autoreactive T cells more strongly potentially explaining their superior EAM induction. GM-cDCs are phenotypically most closely related to *in vivo* cDC2s because they lack the typical cDC1 marker XCR-1 and have low IRF8 and high IRF4 expression (Supplementary Figure 2A). Endogenous *ex vivo* sorted cDC2s from inflamed heart and mLN also displayed superior autoreactive T cell activation and induced polarization toward Th17 and Th1 effector cells. On the contrary, cDC1s also accumulated in the heart and draining mLN, yet failed to induce strong TCR-M activation and polarization. These findings are in line with the general concept that cDC2s are more specialized than cDC1s in presenting antigen to CD4^+^ T cells, due to expression of the antigen processing machinery necessary for MHCII peptide loading, a finding confirmed here with DC subsets from EAM hearts and recently also confirmed by our own study on DCs in the infarcted heart (8, 54).

In this model of EAM induction by BMDCs, initial autoimmunity was induced by repeated immunization with CD40 and LPS-matured BMDCs, yet subsequently, cardiac endogenous cDC2s acquired a capacity to present cardiac self-antigen *ex vivo*, a feature not seen with cDC2s isolated from steady state hearts. The accumulation of cardiac cDCs and MCs in progressing EAM was most likely caused by an amplificatory loop of chemokine production emanating from the cardiac MCs, which produce a large set of CCL chemokines that can recruit DCs and monocytes to the heart, like CCL2 (55, 56). As none of the APC subsets of healthy heart and mLN stimulated TCR-M cells in *ex vivo* co-cultures, it was very likely that upon EAM initiation, endogenous cDC2s in the inflammatory environment took up dying cardiomyocytes and became activated and licensed to prime autoreactive T cells at least *ex vivo*, as shown by the increased expression of T cell co-stimulatory molecules and Th17 polarizing cytokines by the cardiac cDC2s. These data our in accordance with the function of cDC2s in the infarcted heart (8). On the other hand, MCs became competent to produce pro-inflammatory chemokines. At this stage, we can only speculate which signals drive the activation of endogenous cDCs and MCs. In an environment of sterile tissue damage, heart DCs can be activated through danger signals like biglycan and tenascin-C (TC-C) released upon cardiac injury. These danger signals are as potent as microbial TLR ligands (LPS) to stimulate αMyHC loaded BMDCs to induce EAM *in vivo* (20, 21). TC-C KO mice were protected from EAM suggesting that this danger signal is critical for triggering DC activation and subsequent priming and expansion of heart-specific CD4^+^ T cells.

When cultured together with TCR-M cells, sorted cDC2s were able to induce Th1 and Th17 cytokine production, suggesting they might be crucial for induction of cardiac autoimmunity. Heart-infiltrating CD4^+^ T cells in TCR-M mice suffering from spontaneous autoimmune myocarditis, also showed a mixed Th1/Th17 cytokine profile (35). Like the cardiac cDC2s in this study, IRF4-dependent CD103^+^CD11b^+^ cDC2s of the intestinal lamina propria also produced IL-6 and IL-23 and polarized CD4^+^ T cells to produce IL-17 (50). Previous studies have shown that IL-6 and IL-23 deficient mice are resistant to EAM (18, 57), and results from our study now suggest that this might be caused by defective Th17 polarization by cardiac cDC2s. To validate this hypothesis, we set up EAM experiments in *Irf4*^*fl*/*fl*^*.Cd11cCre* mice. These mice have been shown to have defective Th17 immune response induction in mucosal tissues (50, 51), deficits in migration of cDC2s to the draining nodes of skin, lungs and heart (8, 26, 39, 41, 58), and a generalized deficiency in MHCII antigen presentation (42). However, in the EAM model, despite a significant reduction in cDC2 migration to the mLN, *Irf4*^*fl*/*fl*^*.Cd11cCre* mice were not protected from disease, most likely due to the fact that residual IRF4-deficient cDC2s were fully competent in activating TCR-M cells. Autoreactive T cell activation following MI was also intact in *Irf4*^*fl*/*fl*^*.Cd11cCre* mice (8). As cardiac cDC2s were largely unaffected by loss of IRF4 in the EAM model and myocardial infarction, it is also possible that cDC2s or MCs acted locally in the heart to activate effector autoreactive CD4^+^ T cells. We speculated that the injection of WT BMDCs might have bypassed the need for IRF4 in endogenous cDC2s for EAM development. However, we also performed experiments using immunizations with IRF4-deficient BMDCs, and also found EAM induction to be intact (Figure 5C). We cannot explain why we did not see the reduction in MHCII-mediated antigen presentation that was previously reported in IRF4-deficient DCs, except for the fact that previous work showing this association was done using BMDCs, and studying antigen responses in the absence of strong adjuvant systems (42). As we show here in our RNA-seq analysis, there are no good genes that can separate cDC2s from MCs, and until now, no good models exist to selectively and completely delete functional cDC2s.

The MCs accumulating in EAM hearts have been described before, but interpretation of what these cells do varies between authors. Previous work has shown that the NO-producing monocytes and MCs of the heart suppress development of DCM via production of NO and by suppression of T cell expansion (31, 59). As observed in the lung during airway inflammation, we speculate that heart MCs in EAM display a MF-phenotype and are significant producers of chemokines and cytokines, thus controlling influx of inflammatory leukocytes and effector lymphocytes and most likely activating them locally in conjunction with heart cDC2s (36). In support, gene expression profiles of cardiac CD11c^+^ MCs in EAM greatly resembled transcriptome of CD11c^−^ MCs that are often called MFs (Figure 3A). Heart CD11c^+^ MCs produced typical MF cytokines like IL-1α and CCL2, the predominant chemokine controlling influx of effector cells. In a model in which CCL2 was inactivated, EAM severity and progression toward DCM was reduced (60). Here, blockade of the CCL2/CCR2 axis suppressed the downstream chemokine and cytokine cascade, suggesting that CCR2-dependent cells like MCs were crucial in controlling ongoing inflammation. There is certainly a possibility that once EAM is initiated and cardiomyocytes start to die, MCs also present cardiac antigens *in situ* to self-reactive T cells. This is supported by our findings that cardiac MCs were very proficient in presenting self-antigens from dying cardiomyocytes *ex vivo*. In such an *in situ* antigen presenting scenario, there would also be no need for MCs to migrate to the draining nodes, and observation we also made in this study.

In conclusion, we have studied the contribution of various APCs in the onset and progression of EAM, and found that GM-cDC2s, and not GM-MCs, were able to initiate EAM. With progression of disease, endogenous CD11c^+^ cells do not require IRF4 for EAM to develop to its full extent, and several APC subtypes are endowed with APC function. We can only speculate that in humans a similar scenario would be operative. In humans, DC subsets can be reliably identified using a panel of monoclonal antibodies. Our study suggests that is worth analyzing the distribution of DC subsets in the diagnostic endomyocardial biopsies taken from early cases of viral myocarditis. In the future, it might be possible to target the function of cDC2s and MCs in an attempt to dampen myocardial inflammation and progression to DCM.

## Author contributions

BNL and TG: conceptualization; KV, DS, and CS: methodology; LM: formal analysis; GV and YS: investigation; LB, VN, and BL: resources; KV: writing - original draft; CS and BNL: writing - review and editing; BNL and TG: funding acquisition; BNL, TG, and CS: supervision.

### Conflict of interest statement

The authors declare that the research was conducted in the absence of any commercial or financial relationships that could be construed as a potential conflict of interest.

## Supplementary material

Supplementary Figure 1 (related to Figure 1) **(A)** 35 days following BMDC injection, echocardiography was performed and short axis M-mode images are shown of OVA DC injected mice and αMyHC DC injected mice. LVID dias, Left Ventricular Internal Diameter in diastole; LVID sys, Left Ventricular Internal Diameter in systole. **(B)** Graph depicting LVID in systole in groups of mice displayed in panel **A** at day 35 post-immunization. **(C,D)** Ejection fraction (EF) **(C)** and heart/body weight ratio **(D)** of groups of mice displayed in panel A (mean ± SEM; ^**^*P* ≤ 0.01; ^***^*P* ≤ 0.001). **(E)** Survival curve of groups of mice displayed in panel **A**. All bar graphs show data as mean ± SEM; ^**^*P* ≤ 0.01; ^***^*P* ≤ 0.001.

Supplementary Figure 2 (related to Figure 1) **(A)** Flow cytometry gating strategy for sorting of MHCII^hi^PD-L2^+^CD115^−^ GM-cDC2s and MHCII^int^PD-L2^−^CD115^+^ GM-MCs from bulk BMDCs harvested at day 10 of culture. **(B)** Dilution of proliferation dye eF450 and CD25 expression of TCR-M cells co-cultured for 4 days with sorted GM-cDC2s or sorted GM-MCs with addition of dead cardiomyocytes in a 1/10 DC/TCR-M ratio. **(C)** 4 days after naïve TCR-M injection and 3 days after BMDC injection, depicted LNs and spleen were isolated. CFSE dilution and CD25 expression of donor TCR-M cells was analyzed by flow cytometry. **(D)** Percentage of proliferation and CD25 expression of donor TCR-M cells in LNs and spleen from experiment described in **(B)**. **(E)** 4 days after naïve TCR-M injection into steady state mice (not injected with BMDCs), CFSE dilution, and CD25 expression of donor TCR-M cells was analyzed by flow cytometry. All bar graphs show data as mean ± SEM; ^*^*P* ≤ 0.05.

Supplementary Figure 3 (related to Figure 4) **(A,B)** CFSE dilution and CD25 expression of TCR-M cells co-cultured with sorted APC subsets of heart (shown in **A**) and mLN (shown in **B**) at EAM day 10 with addition of 15 μg/ml αMyHC_614−629_ peptide. **(C)** CFSE dilution and CD25 expression of TCR-M cells co-cultured with sorted DC subsets of inguinal LN at EAM day 10.

Supplementary Table 1 List of differentially expressed genes in cDC2s from steady state compared to EAM heart.
