# Supplementary figures and images for: Myocarditis Elicits Dendritic Cell and Monocyte Infiltration in the Heart and Self-Antigen Presentation by Conventional Type 2 Dendritic Cells
Source: Front Immunol. 2018 Nov 21;9:2714. doi: 10.3389/fimmu.2018.02714 (PMC6258766; doi:10.3389/fimmu.2018.02714)

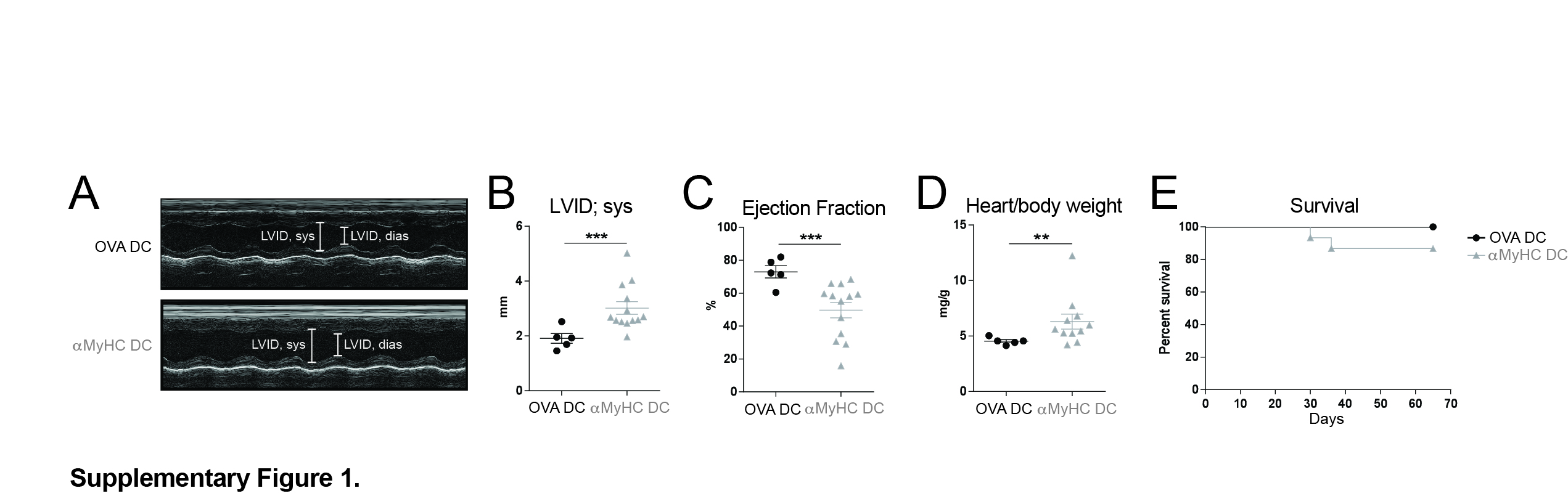

Supplement: Supplementary Figure 1 (related to Figure 1) — (A) 35 days following BMDC injection, echocardiography was performed and short axis M-mode images are shown of OVA DC injected mice and αMyHC DC injected mice. LVID dias, Left Ventricular Internal Diameter in diastole; LVID sys, Left Ventricular Internal Diameter in systole. (B) Graph depicting LVID in systole in groups of mice displayed in panel A at day 35 post-immunization. (C,D) Ejection fraction (EF) (C) and heart/body weight ratio (D) of groups of mice displayed in panel A (mean ± SEM; **P ≤ 0.01; ***P ≤ 0.001). (E) Survival curve of groups of mice displayed in panel A. All bar graphs show data as mean ± SEM; **P ≤ 0.01; ***P ≤ 0.001. [file Image_1.JPEG]

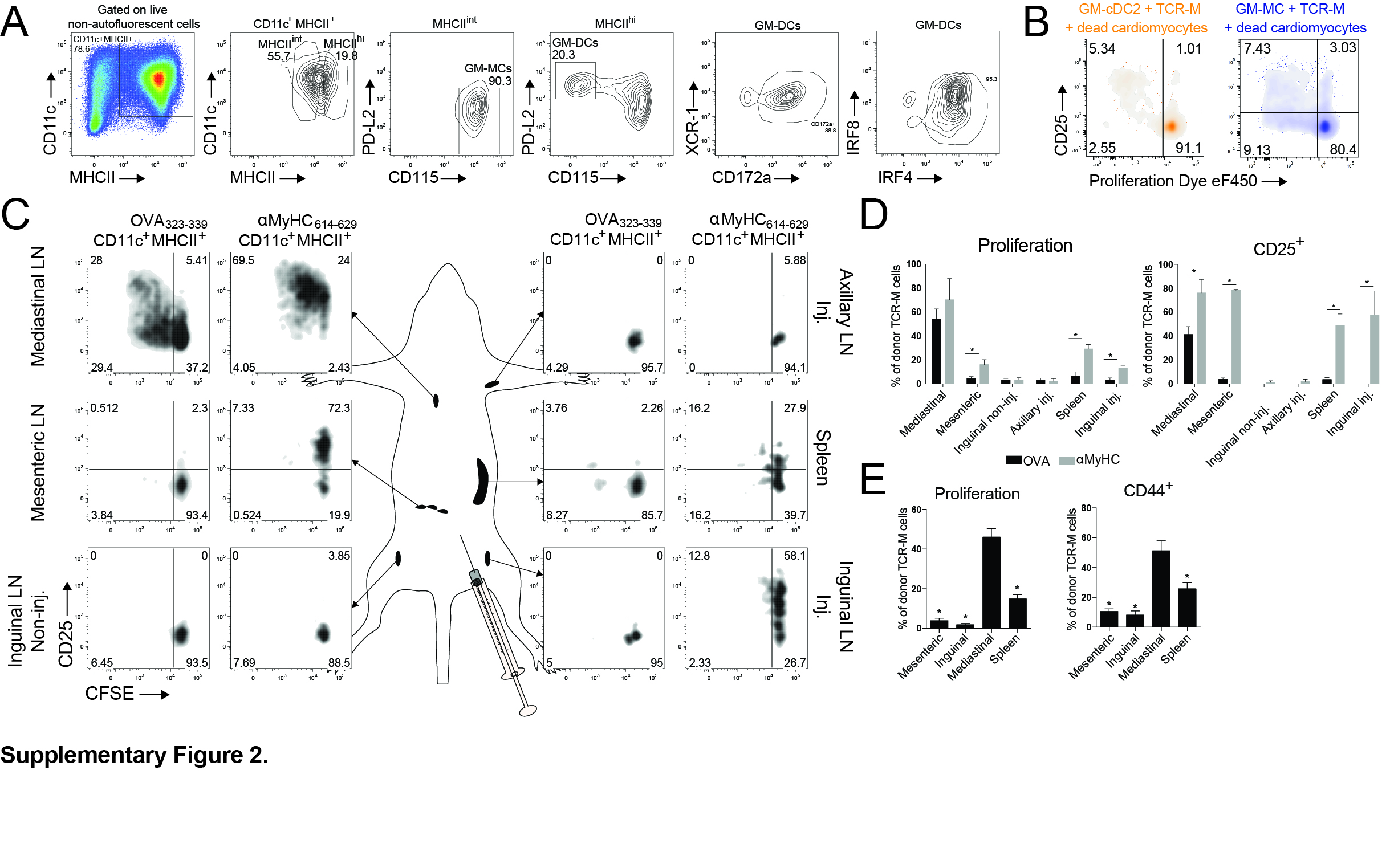

Supplement: Supplementary Figure 2 (related to Figure 1) — (A) Flow cytometry gating strategy for sorting of MHCIIhiPD-L2+CD115− GM-cDC2s and MHCIIintPD-L2−CD115+ GM-MCs from bulk BMDCs harvested at day 10 of culture. (B) Dilution of proliferation dye eF450 and CD25 expression of TCR-M cells co-cultured for 4 days with sorted GM-cDC2s or sorted GM-MCs with addition of dead cardiomyocytes in a 1/10 DC/TCR-M ratio. (C) 4 days after naïve TCR-M injection and 3 days after BMDC injection, depicted LNs and spleen were isolated. CFSE dilution and CD25 expression of donor TCR-M cells was analyzed by flow cytometry. (D) Percentage of proliferation and CD25 expression of donor TCR-M cells in LNs and spleen from experiment described in (B). (E) 4 days after naïve TCR-M injection into steady state mice (not injected with BMDCs), CFSE dilution, and CD25 expression of donor TCR-M cells was analyzed by flow cytometry. All bar graphs show data as mean ± SEM; *P ≤ 0.05. [file Image_2.JPEG]

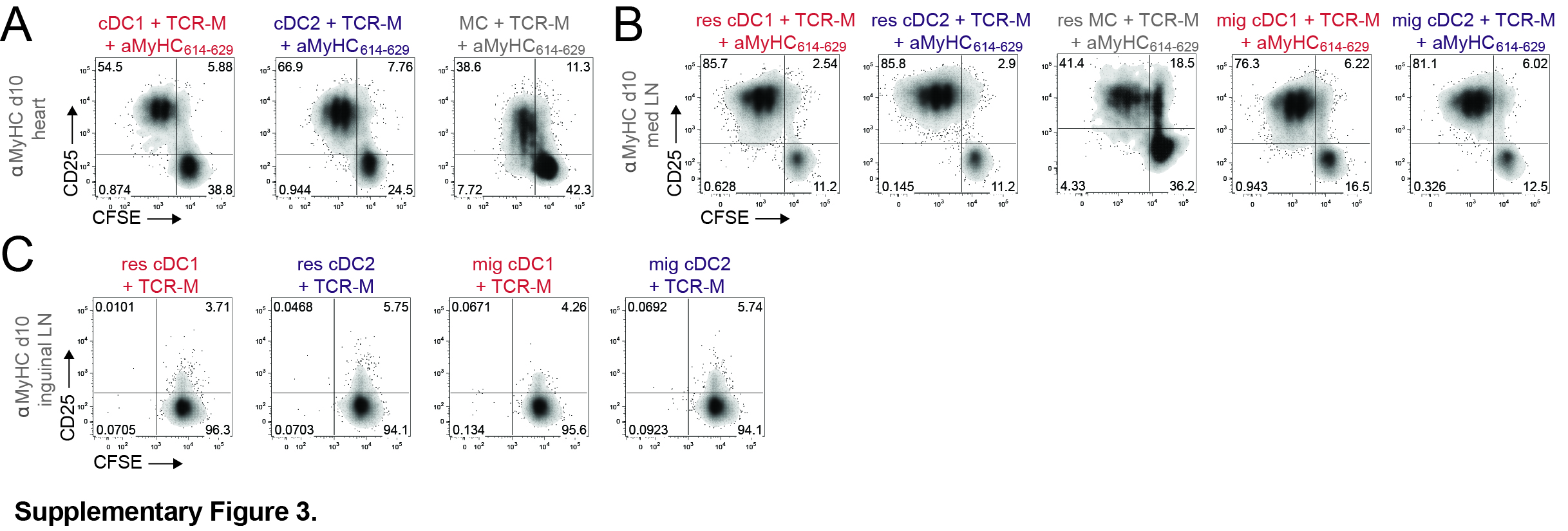

Supplement: Supplementary Figure 3 (related to Figure 4) — (A,B) CFSE dilution and CD25 expression of TCR-M cells co-cultured with sorted APC subsets of heart (shown in A) and mLN (shown in B) at EAM day 10 with addition of 15 μg/ml αMyHC614−629 peptide. (C) CFSE dilution and CD25 expression of TCR-M cells co-cultured with sorted DC subsets of inguinal LN at EAM day 10. [file Image_3.JPEG]
