# Supplementary material for: Myocarditis Elicits Dendritic Cell and Monocyte Infiltration in the Heart and Self-Antigen Presentation by Conventional Type 2 Dendritic Cells
Source: Front Immunol. 2018 Nov 21;9:2714. doi: 10.3389/fimmu.2018.02714 (PMC6258766; doi:10.3389/fimmu.2018.02714)
Supplement: Supplementary file 4 [file Data_Sheet_1.PDF]

Gene symbol	Gene description	Alternative name	Function (related to DCs)	Reference(s)	
Wash1	WAS protein family homolog 1		MHCII recycling	(Graham et al., 2014)	Function in DCs
Irf8	interferon regulatory factor 8	ICSBP	cDC1 development	(Sichien et al., 2016)	
Pacsin1	protein kinase C and casein kinase substrate in neurons 1	Syndapin	type I interferon response in pDCs	(Esashi et al., 2012)	
Serpinb9	serine peptidase inhibitor, clade B, member 9		antigen cross-presentation	(Rizzitelli et al., 2012)	
Plxnc1	plexin C1	CD232	DC migration and mobility	(Walzer et al., 2005)	
Pnkd	paroxysmal nonkinesiogenic dyskinesia	MR-1	NFkB and AP-1 activity	(Dai et al., 2010)	Immune function
Pik3r1	phosphatidylinositol 3-kinase p85 alpha		1-phosphatidylinositol-3-kinase activity	(Hayashi et al., 2017)	
Birc2	baculoviral IAP repeat-containing 2	C-IAP1	innate immune responses	(Prakash et al., 2009)	
Traf3ip1	TRAF3 interacting protein 1	MIP-T3	type I interferon response	(Ng et al., 2011)	
Clic4	chloride intracellular channel 4		LPS signaling	(He et al., 2011)	
Sh3bp4	SH3-domain binding protein 4		Rag GTPase-mTORC1 signaling	(Kim et al., 2012)	
Zfp36l1	zinc finger protein 36, C3H type-like 1		DNA Damage Response Signaling	(Vogel et al., 2016)	
Aebp2	AE binding protein 2		cell migration	(Kim et al., 2015)	Cell migration and division
Csrp1	cysteine and glycine-rich protein 1		cell migration	(Miyasaka et al., 2007)	
Clasp1	CLIP associating protein 1		cell division	(Samora et al., 2011)	
Ccser2	coiled-coil serine rich 2	Gcap14	cell division	(Hosono et al., 2012)	
Kif21b	kinesin family member 21B		cell migration	(Muhia et al., 2016)	
Repin1	replication initiator 1		glucose and lipid metabolism	(Kunath et al., 2016)	Metabolism
Cpt2	carnitine palmitoyltransferase 2		fatty acid metabolic process	(Pereyra et al., 2017)	
Ift172	intraflagellar transport 172		ciliogenesis	(Friedland-Little et al., 2011)	ciliogenesis
Rabep2	rabaptin, RAB GTPase binding effector protein 2		ciliogenesis	(Airik et al., 2016)	
1200014J11Rik	nuclear cap binding subunit 3	Ncbp3	mRNA biogenesis	(Gebhardt et al., 2015)	Miscellaneous
Slc4a8	solute carrier family 4 (anion exchanger), member 8		anion transport	(Wang et al., 2001)	
Cul9	cullin 9		cell apoptosis	(Li and Xiong, 2017)	
Ssh1	slingshot protein phosphatase 1		actin cytoskeleton organization	(Kurita et al., 2007)	
Sept3	septin 3		DC related function(s) unknown		
Tmem120b	transmembrane protein 120B				
Ccdc6	coiled-coil domain containing 6				
Basp1	brain abundant, membrane attached signal protein 1	CAP23			
Gstt3	glutathione S-transferase, theta 3				
Cxcr2	C-X-C chemokine receptor type 2				
Coq4	coenzyme Q4				
Mink1	misshapen-like kinase 1				
Rcl1	RNA terminal phosphate cyclase-like 1				
Mif4gd	MIF4G domain containing				
Tmem39a	transmembrane protein 39a		Molecular function unknown		
Dhx57	DEAH box polypeptide 57				
AI987944	expressed sequence AI987944				
Fam132a	family with sequence similarity 132, member A				
Wdr86	WD repeat domain 86				
Sdr39u1	short chain dehydrogenase/reductase family 39U, member 1				

Supplementary Table 1.
