# Supplementary material for: Myocarditis Elicits Dendritic Cell and Monocyte Infiltration in the Heart and Self-Antigen Presentation by Conventional Type 2 Dendritic Cells
Source: Front Immunol. 2018 Nov 21;9:2714. doi: 10.3389/fimmu.2018.02714 (PMC6258766; doi:10.3389/fimmu.2018.02714)
Supplement: Supplementary Table 1 — List of differentially expressed genes in cDC2s from steady state compared to EAM heart. [file Data_Sheet_2.PDF]

Reference list Table S1

Airik, R., Schueler, M., Airik, M., Cho, J., Ulanowicz, K.A., Porath, J.D., Hurd, T.W., Bekker-Jensen, S., Schroder, J.M., Andersen, J.S., and Hildebrandt, F. (2016). SDCCAG8 Interacts with RAB Effector Proteins RABEP2 and ERC1 and Is Required for Hedgehog Signaling. *PLoS One* 11**,** e0156081.

Dai, W., Chen, H., Jiang, J., Kong, W., and Wang, Y. (2010). Silencing MR-1 attenuates inflammatory damage in mice heart induced by AngII. *Biochem Biophys Res Commun* 391**,** 1573-1578.

Esashi, E., Bao, M., Wang, Y.H., Cao, W., and Liu, Y.J. (2012). PACSIN1 regulates the TLR7/9-mediated type I interferon response in plasmacytoid dendritic cells. *Eur J Immunol* 42**,** 573-579.

Friedland-Little, J.M., Hoffmann, A.D., Ocbina, P.J., Peterson, M.A., Bosman, J.D., Chen, Y., Cheng, S.Y., Anderson, K.V., and Moskowitz, I.P. (2011). A novel murine allele of Intraflagellar Transport Protein 172 causes a syndrome including VACTERL-like features with hydrocephalus. *Hum Mol Genet* 20**,** 3725-3737.

Gebhardt, A., Habjan, M., Benda, C., Meiler, A., Haas, D.A., Hein, M.Y., Mann, A., Mann, M., Habermann, B., and Pichlmair, A. (2015). mRNA export through an additional cap-binding complex consisting of NCBP1 and NCBP3. *Nat Commun* 6**,** 8192.

Graham, D.B., Osborne, D.G., Piotrowski, J.T., Gomez, T.S., Gmyrek, G.B., Akilesh, H.M., Dani, A., Billadeau, D.D., and Swat, W. (2014). Dendritic cells utilize the evolutionarily conserved WASH and retromer complexes to promote MHCII recycling and helper T cell priming. *PLoS One* 9**,** e98606.

Hayashi, S., Hamada, T., Zinsou, D.G.A., Oshiro, M., Itoi, K., Yamamoto, T., and Kadowaki, M. (2017). PI3K p85alpha Subunit-deficient Macrophages Protect Mice from Acute Colitis due to the Enhancement of IL-10 Production. *Sci Rep* 7**,** 6187.

He, G., Ma, Y., Chou, S.Y., Li, H., Yang, C., Chuang, J.Z., Sung, C.H., and Ding, A. (2011). Role of CLIC4 in the host innate responses to bacterial lipopolysaccharide. *Eur J Immunol* 41**,** 1221-1230.

Hosono, H., Yamaguchi, N., Oshima, K., Matsuda, T., and Nadano, D. (2012). The murine Gcap14 gene encodes a novel microtubule binding and bundling protein. *FEBS Lett* 586**,** 1426-1430.

Kim, H., Ekram, M.B., Bakshi, A., and Kim, J. (2015). AEBP2 as a transcriptional activator and its role in cell migration. *Genomics* 105**,** 108-115.

Kim, Y.M., Stone, M., Hwang, T.H., Kim, Y.G., Dunlevy, J.R., Griffin, T.J., and Kim, D.H. (2012). SH3BP4 is a negative regulator of amino acid-Rag GTPase-mTORC1 signaling. *Mol Cell* 46**,** 833-846.

Kunath, A., Hesselbarth, N., Gericke, M., Kern, M., Dommel, S., Kovacs, P., Stumvoll, M., Bluher, M., and Kloting, N. (2016). Repin1 deficiency improves insulin sensitivity and glucose metabolism in db/db mice by reducing adipose tissue mass and inflammation. *Biochem Biophys Res Commun* 478**,** 398-402.

Kurita, S., Gunji, E., Ohashi, K., and Mizuno, K. (2007). Actin filaments-stabilizing and -bundling activities of cofilin-phosphatase Slingshot-1. *Genes Cells* 12**,** 663-676.

Li, Z., and Xiong, Y. (2017). Cytoplasmic E3 ubiquitin ligase CUL9 controls cell proliferation, senescence, apoptosis and genome integrity through p53. *Oncogene* 36**,** 5212-5218.

Miyasaka, K.Y., Kida, Y.S., Sato, T., Minami, M., and Ogura, T. (2007). Csrp1 regulates dynamic cell movements of the mesendoderm and cardiac mesoderm through interactions with Dishevelled and Diversin. *Proc Natl Acad Sci U S A* 104**,** 11274-11279.

Muhia, M., Thies, E., Labonte, D., Ghiretti, A.E., Gromova, K.V., Xompero, F., Lappe-Siefke, C., Hermans-Borgmeyer, I., Kuhl, D., Schweizer, M., Ohana, O., Schwarz, J.R., Holzbaur, E.L.F., and Kneussel, M. (2016). The Kinesin KIF21B Regulates Microtubule Dynamics and Is Essential for Neuronal Morphology, Synapse Function, and Learning and Memory. *Cell Rep* 15**,** 968-977.

Ng, M.H., Ho, T.H., Kok, K.H., Siu, K.L., Li, J., and Jin, D.Y. (2011). MIP-T3 is a negative regulator of innate type I IFN response. *J Immunol* 187**,** 6473-6482.

Pereyra, A.S., Hasek, L.Y., Harris, K.L., Berman, A.G., Damen, F.W., Goergen, C.J., and Ellis, J.M. (2017). Loss of cardiac carnitine palmitoyltransferase 2 results in rapamycin-resistant, acetylation-independent hypertrophy. *J Biol Chem* 292**,** 18443-18456.

Prakash, H., Becker, D., Bohme, L., Albert, L., Witzenrath, M., Rosseau, S., Meyer, T.F., and Rudel, T. (2009). cIAP-1 controls innate immunity to C. pneumoniae pulmonary infection. *PLoS One* 4**,** e6519.

Rizzitelli, A., Meuter, S., Vega Ramos, J., Bird, C.H., Mintern, J.D., Mangan, M.S., Villadangos, J., and Bird, P.I. (2012). Serpinb9 (Spi6)-deficient mice are impaired in dendritic cell-mediated antigen cross-presentation. *Immunol Cell Biol* 90**,** 841-851.

Samora, C.P., Mogessie, B., Conway, L., Ross, J.L., Straube, A., and Mcainsh, A.D. (2011). MAP4 and CLASP1 operate as a safety mechanism to maintain a stable spindle position in mitosis. *Nat Cell Biol* 13**,** 1040-1050.

Sichien, D., Scott, C.L., Martens, L., Vanderkerken, M., Van Gassen, S., Plantinga, M., Joeris, T., De Prijck, S., Vanhoutte, L., Vanheerswynghels, M., Van Isterdael, G., Toussaint, W., Madeira, F.B., Vergote, K., Agace, W.W., Clausen, B.E., Hammad, H., Dalod, M., Saeys, Y., Lambrecht, B.N., and Guilliams, M. (2016). IRF8 Transcription Factor Controls Survival and Function of Terminally Differentiated Conventional and Plasmacytoid Dendritic Cells, Respectively. *Immunity* 45**,** 626-640.

Vogel, K.U., Bell, L.S., Galloway, A., Ahlfors, H., and Turner, M. (2016). The RNA-Binding Proteins Zfp36l1 and Zfp36l2 Enforce the Thymic beta-Selection Checkpoint by Limiting DNA Damage Response Signaling and Cell Cycle Progression. *J Immunol* 197**,** 2673-2685.

Walzer, T., Galibert, L., and De Smedt, T. (2005). Dendritic cell function in mice lacking Plexin C1. *Int Immunol* 17**,** 943-950.

Wang, Z., Conforti, L., Petrovic, S., Amlal, H., Burnham, C.E., and Soleimani, M. (2001). Mouse Na+: HCO3- cotransporter isoform NBC-3 (kNBC-3): cloning, expression, and renal distribution. *Kidney Int* 59**,** 1405-1414.
